# Nail Lichen Planus in 81 Patients: A Retrospective Study of Clinical Characteristics, Histopathological Features, and Long‐Term Treatment Outcomes

**DOI:** 10.1111/1346-8138.17907

**Published:** 2025-08-14

**Authors:** Juan He, Tengyu Weng, Jia Bai, Wenwei Zhu, Xingyu Huang, Yi Yang, Chengxin Li

**Affiliations:** ^1^ School of Medicine Nankai University Tianjin China; ^2^ Department of Dermatology The First Medical Center of Chinese PLA General Hospital Beijing China; ^3^ Department of Dermatology The Third Medical Center of Chinese PLA General Hospital Beijing China

**Keywords:** clinical feature, histopathological feature, nail lichen planus, treatment outcomes

## Abstract

Nail lichen planus (NLP) is a rare, chronic inflammatory nail disorder that can result in irreversible damage. While clinical awareness is growing, long‐term real‐world data—particularly incorporating histopathological confirmation and treatment outcomes—remains limited. We retrospectively analyzed 81 biopsy‐confirmed NLP cases (2016–2024) from a Chinese dermatology center to characterize their epidemiological, clinical, histological features, treatment responses, and long‐term outcomes. The mean age at onset of our cohort was 44.41 ± 17.77 years, with a slight male predominance (59.26%). Nail matrix involvement occurred in 97.53% of patients, and disease severity did not correlate with disease duration. Longitudinal biopsies showed a 96.30% concordance rate between clinical and histopathologic localization and revealed ventral proximal nail fold inflammation in 58.02% of patients. Patient satisfaction with the biopsy procedure was generally high (mean score: 8.32), though 14.52% reported dissatisfaction due to post‐procedure deformity. Misdiagnosis occurred in 44.44% of patients. Among 62 patients with treatment follow‐up, systemic corticosteroids were the most effective therapy, especially in those with periungual erythema; although 42.86% relapsed after discontinuation. JAK inhibitors demonstrated encouraging results in refractory cases. Oral acitretin showed moderate efficacy in early disease, while topical corticosteroids, calcineurin inhibitors, and hydroxychloroquine were largely ineffective. This study presents the largest long‐term cohort of biopsy‐confirmed NLP to date and offers new insights into its clinical spectrum, diagnostic strategies, and real‐world treatment outcomes. It highlights the utility and limitations of longitudinal biopsy, the potential predictive value of periungual inflammation, and the emerging role of JAK inhibitors as promising therapeutic agents in recalcitrant cases.

## 1 Introduction

**FIGURE 1:**
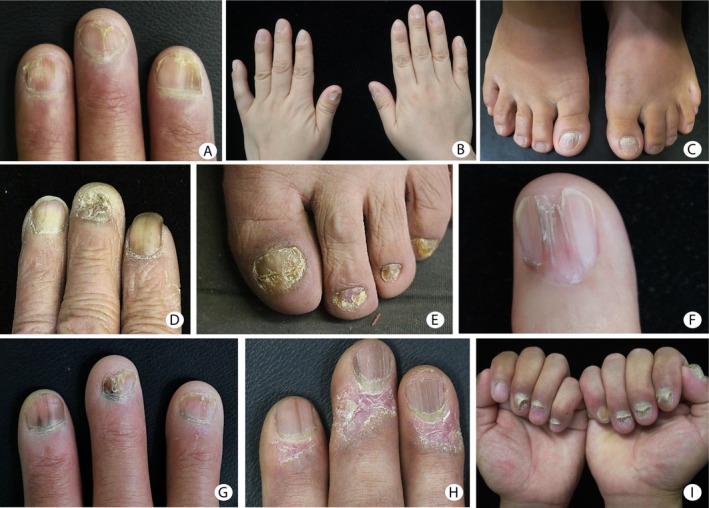
Representative clinical manifestations of nail lichen planus (NLP) across its subtypes and stages. (A) Classical NLP: Plate thinning with longitudinal ridging and splitting, with or without early pterygium formation. (B, C) Twenty‐nail dystrophy type NLP: Uniform and monomorphic appearance characterized by diffuse longitudinal ridging involving all fingernails, typically without pterygium. (D, E) Idiopathic atrophy type NLP: Marked nail plate atrophy affecting multiple nails, with variable presence of nail plate loss and pterygium formation. (F) Advanced pterygium formation: A fan‐shaped extension of the proximal nail fold onto the nail bed, replacing the nail matrix with scar tissue, leading to irreversible nail loss. (G) Clinical signs of matrix and nail bed involvement in NLP: Features include longitudinal melanonychia, plate splitting, onychoschizia, and erythronychia. Nail bed involvement is indicated by onycholysis. (H) Severe matrix‐predominant NLP: Pronounced nail thinning, longitudinal melanonychia, and marked erythema with scaling of the proximal nail fold. (I) Nail bed–predominant NLP: Extensive onycholysis and subungual hyperkeratosis without significant matrix changes.

Lichen planus (LP), first described by Erasmus Wilson in 1869, is a chronic inflammatory dermatosis that can affect the skin, mucous membranes, hair follicles, and nails [1]. While nail lichen planus (NLP), first described in the early 20th century [2], is estimated to occur in approximately 10%–15% of patients with LP [3]. Although less common than cutaneous or mucosal forms, NLP can lead to significant functional and cosmetic impairment, particularly in advanced stages [4]. Clinically, NLP presents in three major forms: typical NLP, 20‐nail dystrophy, and idiopathic atrophy of the nails [5] (Figure 1A–E).

Despite growing awareness of NLP, comprehensive long‐term data on its treatment responses and prognosis remain scarce [5, 6, 7, 8, 9]. Hence, we retrospectively analyzed 81 patients with biopsy‐confirmed NLP from a dermatology center, providing detailed characterization of their clinical features, histopathologic patterns, treatment responses, and long‐term follow‐up data.

## 2 Methods

### 2.1 Study Design and Participants

We conducted a retrospective study of patients diagnosed with isolated NLP from June 2016 to June 2024 at the Department of Dermatology of First Medical Center of PLA General Hospital. Diagnosis of NLP was established based on characteristic clinical features and supported by histopathological confirmation.

Typical clinical features of NLP include nail matrix changes (e.g., longitudinal ridging, splitting, thinning, chromonychia), nail bed involvement (e.g., onycholysis, subungual hyperkeratosis), nail fold inflammation (e.g., erythema, scaling), and irreversible damage such as dorsal pterygium and anonychia.

Histopathologic diagnosis was made when nail unit biopsy demonstrated a lichenoid interface dermatitis pattern with features such as basal keratinocyte degeneration, a band‐like lymphocytic infiltrate, and hypergranulosis. In late‐stage NLP, histologic changes may be less specific and include disappearance of the nail matrix, replacement of normal epithelial structures with scar tissue, and loss of inflammatory infiltrate. Inclusion criteria were NLP patients confirmed by longitudinal nail biopsy. Patients with incomplete clinical or histopathologic data were excluded. Of the 89 patients screened, 8 patients with incomplete data were excluded, and 81 patients were enrolled. The study received approval from the ethical committee (S2023‐179) of the Chinese PLA General Hospital and adhered to the ethical guidelines at our institution, following the principles outlined in the Helsinki Declaration.

### 2.2 Clinical Data Collection

Clinical data included age, gender, disease duration, potential triggering factors, family history, presence of other lichen planus variants, and coexistence of other autoimmune disorders. Nail involvement was assessed by the number and location of affected nails (fingernails, toenails), associated symptoms, and NLP subtype. Clinical nail presentations were classified anatomically (Figure 1F–I): nail matrix involvement lesions included longitudinal ridging/splitting, onychoschizia, nail plate thinning, leukonychia, melanonychia, erythronychia, red lunula, pitting, pterygium, and atrophy. Nail bed involvement lesions comprised onycholysis, subungual hyperkeratosis, and splinter hemorrhages. Nail fold changes included erythema, hypertrophy, or scale lesions in the nail fold. Therapeutic regimens and follow‐up data were recorded.

### 2.3 The Definition of NLP Subtypes

Typical nail lichen planus (NLP) is the most common subtype, clinically characterized by thinning of the nail plate accompanied by longitudinal ridging and splitting, which may be associated with or without pterygium (Figure 1A). Twenty‐nail dystrophy (trachyonychia) presents as a uniform roughness of all nails due to excessive longitudinal ridging, differing from typical NLP by its consistent nail surface involvement and the lack of pterygium (Figure 1B,C). Idiopathic nail atrophy is defined by progressive atrophy affecting multiple nails, with or without nail plate loss or pterygium. Unlike the gradual evolution of typical NLP with pterygium, idiopathic atrophy often shows a more abrupt onset, widespread scarring, and nail loss either from matrix destruction or extensive atrophy, irrespective of pterygium formation (Figure 1D,E).

### 2.4 Nail Biopsy and Histopathology

**FIGURE 2:**
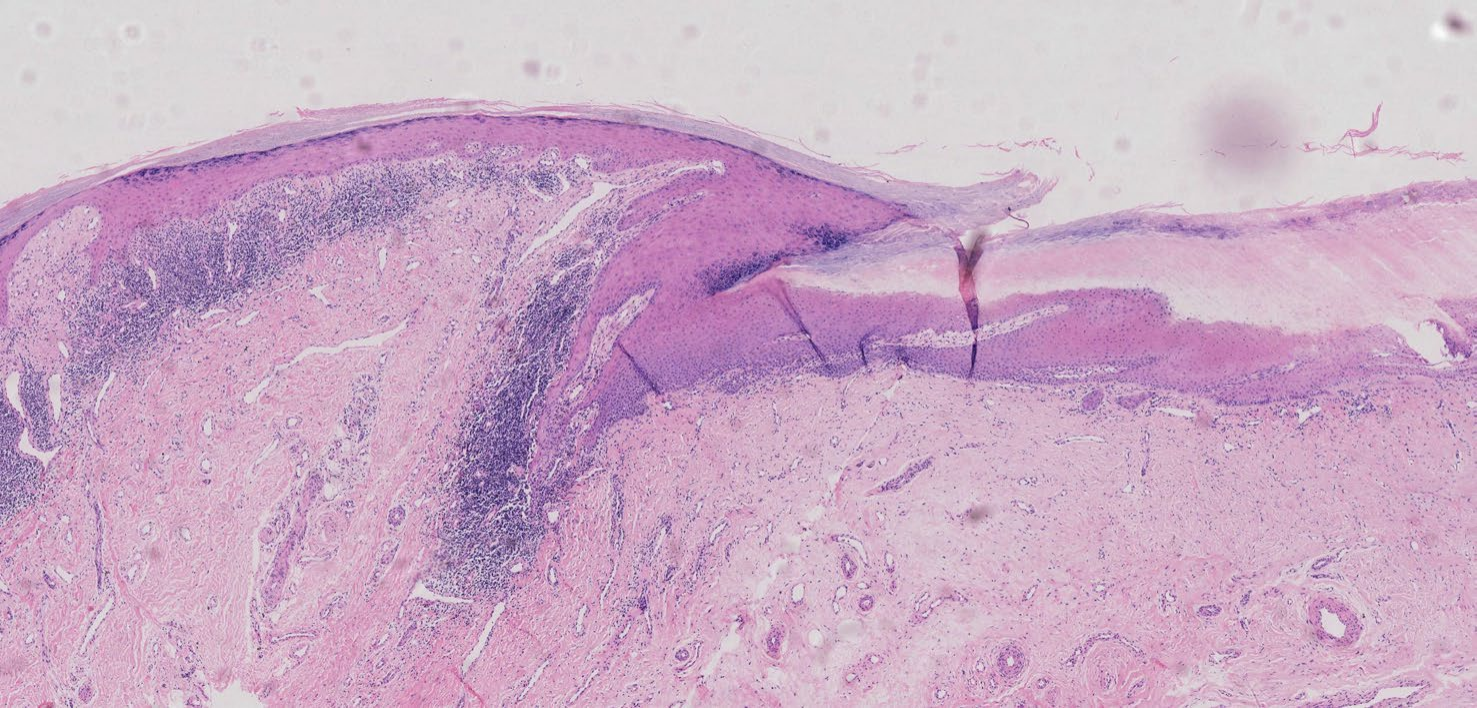
Histopathological findings from longitudinal nail biopsies in nail lichen planus. Longitudinal nail biopsy from a 68‐year‐old woman with NLP demonstrates key histopathological features, including hyperkeratosis, hypergranulosis, and focal lichenoid inflammation involving the dorsal nail fold, ventral nail fold, and nail matrix. Notably, destruction and loss of the keratogenous zone within the matrix are observed, reflecting advanced matrix involvement(HE, 50X).

Longitudinal nail biopsies were performed under digital nerve block using 1% lidocaine without epinephrine. The most serious nail was selected, and a longitudinal biopsy was obtained after partial or complete nail plate avulsion, including the proximal nail fold, matrix, bed, and hyponychium. Specimens were fixed in 10% formalin and stained with H&E. Histopathology consistent with NLP typically shows hyperkeratosis, acanthosis, elongation of rete ridges, a band of lymphocytes in the upper dermis and dermoepidermal junction, and necrosis of basal layer cells in the proximal nail fold, similar to cutaneous LP (Figure 2) [10]. As NLP progresses to late stages, histopathology typically reveals nonspecific findings such as matrix disappearance, epithelial scarring, and diminished inflammatory activity.

### 2.5 The Definition of Disease Severity

NLP severity was categorized as mild, moderate, or severe based on clinical criteria [3]. Mild NLP was defined by features such as nail plate thinning, longitudinal ridging, distal splitting < 3 mm, onycholysis affecting < 25% of the nail surface, and absence of subungual hyperkeratosis. Moderate NLP was characterized by partial fissuring, longitudinal grooves, distal splitting between 3 and 5 mm, onycholysis involving 25%–50% of the nail surface, mottled erythema of the lunula, and the presence of subungual hyperkeratosis. Severe NLP was defined by complete fissuring, deep longitudinal grooves, splitting > 5 mm, onycholysis > 50% of the nail surface, and diffuse erythema of the lunula, pterygium, and/or partial to total anonychia or atrophy.

### 2.6 Definition of Treatment Response

Treatment outcomes were evaluated retrospectively by two senior board‐certified dermatologists with over 10 years of clinical experience in nail disorders. The degree of improvement was based on the physicians' overall clinical impression of changes in nail severity, drawn from standardized clinical photographs and medical records. Outcomes were categorized as follows [3]: No improvement or worsening was defined as a 0% reduction in disease severity; Minimal improvement corresponded to a reduction of ≤ 25%; Mild improvement was defined as a 26%–50% reduction; Moderate improvement as a 51%–75% reduction; Great improvement as a 76%–99% reduction; Clinical cure was defined as complete resolution, with a 100% reduction in disease severity.

### 2.7 Statistical Analysis

Data were analyzed using SPSS 23.0 software (IBM, Armonk, NY, USA). Continuous variables were expressed as mean ± SD or median (range), and categorical variables as frequencies and percentages. Between‐group comparisons used Student's *t*‐test or Mann–Whitney *U* test for continuous variables and chi‐square or Fisher's exact test for categorical variables. A *p*‐value < 0.05 was considered statistically significant.

## 3 Results

### 3.1 Epidemiological Features

Patients affected by NLP had a mean age of 44.41 ± 17.77 years, ranging from 6 years old to 75 years old. The group showed a slight male predominance, with 48 males and 33 females, resulting in a sex ratio of approximately 1.45. Pediatric cases, defined as patients younger than 18 years, comprised 11.11% (9 patients) of the studied cohort. None of the patients reported a family history of LP or identifiable triggers preceding the disease onset. The average time from disease onset to diagnosis was 47.70 ± 54.06 months, with a range spanning from 2 months to 20 years.

### 3.2 Nail Distribution and Subtypes

Clinical examination revealed that an average of 8.70 ± 5.58 nails was involved per patient (range 1–20 nails). Fingernails were the primary site of involvement in 97.53% of cases (79 cases), with 69.14% (56 cases) exclusively involving fingernails. Toenail involvement was observed in 32.10% (26 cases), with isolated toenail NLP occurring in only 2 cases (2.47%). Disease limited to a single fingernail was observed in 8 cases (9.88%). Symmetry in nail involvement was found in 64 cases (79.01%), whereas asymmetrical involvement occurred in 17 cases (20.99%). Regarding NLP subtypes, classical NLP was observed in 58 patients (71.60%), 20‐nail dystrophy in 8 patients (9.88%), and nail atrophy in 15 patients (18.52%). Clinical characteristics of our patients were summarized on Table 1.

**TABLE 1:** Baseline characteristics of 81 patients with nail lichen planus.

Age	
Onset age, year (range)	44.41 ± 17.77 (6–75)
< 18	9 (11.11%)
≥ 18	72 (88.89%)
Sex	
Male	48 (59.26%)
Female	33 (40.74%)
Duration of disease, months	47.70 ± 54.06 months (2 months–20 years)
Affecting nails	8.70 ± 5.58 (1–20)
Subtype of NLP	
Classical	58 (71.60%)
20‐nail dystrophy	8 (9.88%)
Atrophy	15 (18.52%)
Clinical symptoms	
Itching	12 (14.81%)
Pain	11 (13.58%)
Swelling	2 (2.47%)
Concomitant other variant LP	
Cutaneous LP	8 (9.88%)
Mucosal LP	6 (7.41%)
Follicular LP, or lichen planopilaris (LPP)	1 (1.23%)
Concomitant other autoimmune disorders	
Rheumatic immune disease	2 (2.47%)
Severity of clinical presentation	
Mild	10 (12.35%)
Moderate	21 (25.93%)
Severe	50 (61.73%)

Abbreviations: NLP, Nail lichen planus; LP, Lichen planus.

### 3.3 Clinical Features

Clinical examination revealed that 79 out of 81 patients (97.53%) exhibited nail matrix involvement. The most commonly presented as longitudinal ridging (*n* = 56, 69.14%), longitudinal splitting (*n* = 39, 48.15%), and nail thinning (*n* = 35, 43.21%). Additional matrix changes included erythronychia (*n* = 24, 29.63%), true leukonychia (*n* = 16, 19.75%), onychoschizia (*n* = 14, 17.28%), red lunula (*n* = 10, 12.35%), melanonychia (*n* = 8, 9.88%) and nail pitting (*n* = 4, 4.94%). Late‐stage features such as dorsal pterygium (*n* = 6, 7.41%) and nail atrophy (*n* = 15, 18.52%) were also observed. Nail bed changes, observed in 29.63% (*n* = 24) of cases, primarily consisted of subungual hyperkeratosis (*n* = 16, 19.75%), onycholysis (*n* = 12, 14.81%) and splinter hemorrhages (*n* = 10, 12.35%). While periungual inflammation lesions were noted in 17.28% (*n* = 14) of patients (Table 2). Symptoms including itching (*n* = 12, 14.81%), pain (*n* = 11, 13.58%) and swelling (*n* = 2, 2.47%) were occasionally reported. Coexisting mucosal, cutaneous, or follicular LP variants and autoimmune comorbidities were rare, occurring as cutaneous LP in 8 patients (9.88%), mucosal LP in 6 patients (7.41%), and follicular LP in 1 patient (1.23%) (Table 1).

**TABLE 2:** The clinical features of 81 patients with nail lichen planus.

Clinical findings	Number of patients (%)
Nail matrix involvement	79 (97.53%)
Longitudinal ridging	56 (69.14%)
Longitudinal splitting	39 (48.15%)
Onychoschizia	14 (17.28%)
Nail plate thinning	35 (43.21%)
True Leukonychia	16 (19.75%)
Melanonychia	8 (9.88%)
Erythronychia/Red lunula	24 (29.63%)/10 (12.35%)
Nail pitting	4 (4.94%)
Dorsal pterygium	6 (7.41%)
Atrophy	15 (18.52%)
Nail bed involvement	24 (29.63%)
Subungual hyperkeratosis	16 (19.75%)
Onycholysis	12 (14.81%)
Splinter hemorrhage	10 (12.35%)
Nail fold involvement	—
Periungual lesions	14 (17.28%)

### 3.4 The Correlation of Severity With Disease Duration

Among the 81 patients, clinical severity was classified as mild in 10 cases (12.35%), moderate in 21 cases (25.93%), and severe in 50 cases (61.73%). Notably, within the severe group, 15 cases showed advanced nail atrophy (Figure 1D,E) and 6 patients exhibited dorsal pterygium (Figure 1F). The mean disease duration in patients with severe presentation was 54.06 ± 65.18 months (range 3–240 months), compared to 38.45 ± 35.51 months (range 2–108 months) in those with mild to moderate disease. Although the severe group tended to have a longer disease course, the difference between the two groups did not reach statistical significance (*p* = 0.431, unpaired *t*‐test).

### 3.5 Histological Features

All cases were confirmed through longitudinal nail biopsies, showing hyperkeratosis, acanthosis, elongation of rete ridges, a band of lymphocytes in the upper dermis and dermoepidermal junction, and necrosis of basal layer cells in the proximal nail fold, similar to cutaneous LP (Figure 2). Disappearance of the keratogenous zone and hypergranulosis is typically seen in the nail matrix [10] (Table 3). Among the 81 biopsied nails, clinical involvement and histopathological involvement were analyzed; the overall concordance rate between clinical and histopathologic localization was approximately 96.30% (78/81). Interestingly, because longitudinal biopsies allow visualization of the entire nail unit, we found that 47 cases (58.02%) showed pathological involvement of the ventral nail fold, which is often not clinically apparent, especially at the junction between the ventral nail fold and the proximal nail matrix (Figure 2). Moreover, to evaluate the patient‐reported outcomes of the biopsy procedure, we assessed satisfaction with longitudinal nail biopsy in 62 followed‐up patients using a 0–10 scale (10 indicating very satisfied, 0 indicating very dissatisfied). The mean satisfaction score was 8.32 ± 2.86 (range: 1–10). However, it is worth noting that 14.52% of patients (*n* = 9) reported a satisfaction score below 6, mainly due to postoperative nail deformity.

**TABLE 3:** The histopathological features of 81 patients with nail lichen planus.

Histopathological features	Number of dorsal nail fold involvement (*n* = 14)	Number of ventral nail fold involvement (*n* = 47)	Number of nail matrix involvement (*n* = 78)	Number of nail bed involvement (*n* = 22)
Hyperkeratosis/Parakeratosis	4 (28.57%)	6 (12.77%)	17 (21.79%)	1 (4.55%)
Hypergranulosis	8 (57.14%)	24 (51.06%)	40 (51.28%)	12 (54.55%)
Acanthosis	10 (71.43%)	29 (61.70%)	23 (29.49%)	9 (40.91%)
Elongation of rete ridges	7 (50.00%)	21 (44.68%)	34 (43.59%)	8 (36.36%)
Lichenoid band abutting the epithelium	13 (92.86%)	40 (85.11%)	45 (57.69%)	12 (54.55%)
Focal vacuolar degeneration of the basement membrane zone	2 (14.29%)	8 (17.02%)	11 (14.10%)	3 (13.64%)
Colloid bodies	2 (14.29%)	1 (2.13%)	4 (5.13%)	0 (0%)
Partial or total destruction of the keratogenous zone	—	—	23 (29.49%)	—

### 3.6 Treatment and Follow‐Up

A notable finding was that 44.44% of patients (36/81) experienced prior misdiagnosis and mistreatment. These patients had previously been misdiagnosed with onychomycosis (*n* = 20), nail dystrophy (*n* = 11), psoriatic nail disease (*n* = 6), or eczematous nail changes (*n* = 5). Among the 20 misdiagnosed with onychomycosis, 11 patients (55%) had received systemic antifungal treatment such as itraconazole or terbinafine, without clinical improvement. After diagnosis, 62 patients completed long‐term follow‐up and were included in treatment response analysis (Table 4). The average follow‐up duration for this cohort was 5.10 ± 2.41 years, ranging from 11 months to 9 years.

**TABLE 4:** Clinical response distribution to various therapies in nail lichen planus.

Therapy methods	Number of patients	No improvement 0%	Minimal improvement ≤ 25%	Mild improvement (26% to 50%)	Moderate improvement (51% to 75%)	Great improvement (76% to 99%)	Clinical cure (100%)
No therapy	*N* = 6	3	0	0	0	0	3
Topical therapy only	*N* = 9	3	5	1	0	0	0
System treatment	*N* = 47	8	3	3	5	9	19
Acitretin	*N* = 10	2	2	1	0	2	3
Hydroxychloroquine	*N* = 4	4	0	0	0	0	0
Intramuscular triamcinolone	*N* = 18	0	0	2	4	4	8
Oral steroids	*N* = 12	2	1	0	1	2	6
Jak inhibitor	*N* = 3	0	0	0	0	1	2

### 3.7 No Therapy

After 2 to 3 years, 3 patients with moderate 20‐nail dystrophy showed spontaneous remission; 3 patients with severe atrophic‐type NLP experienced further disease progression.

### 3.8 Topical Therapy

Nine patients only received topical treatments, including halometasone, halometasone‐triclosan cream, and tacrolimus ointment. All had classical NLP, with severities ranging from mild to severe. Of these, 3 patients showed no clinical improvement, and 5 showed minimal improvement within 3 to 6 months treatment. Only 1 patient who presented with marked periungual inflammation achieved moderate improvement after 6 months topical therapy. Overall, topical monotherapy yielded limited efficacy. No systemic adverse events were reported. However, mild local side effects were observed in 2 patients, presenting as subtle atrophy of the proximal nail fold after prolonged use of topical corticosteroids.

### 3.9 Systemic Therapies

#### 3.9.1 Oral Acitretin

Ten patients were treated with oral acitretin. Two patients with severe atrophic NLP showed no response. Two patients with moderate classical NLP achieved only minimal improvement, and 1 patient with moderate classical NLP achieved great improvement after 3 months of treatment, but the drug was stopped due to intolerable mucosal dryness and elevated liver enzymes; the disease relapsed after drug withdrawal. Meanwhile, the other 5 patients with mild classical NLP reached great improvement and clinical cure after 6 months to 2 years of continuous treatment. These findings suggest acitretin may be more effective in early‐stage NLP. In terms of safety, the most frequently observed adverse events associated with acitretin included cheilitis, xerosis, and mucocutaneous dryness. No serious long‐term systemic adverse events were reported during follow‐up.

#### 3.9.2 Hydroxychloroquine

Four patients treated with hydroxychloroquine, including three with moderate and one with severe classical NLP, failed to show meaningful improvement. There were no systemic adverse events reported.

#### 3.9.3 Systemic Corticosteroids

Systemic corticosteroid therapy was administered to 30 patients with moderate‐to‐severe classical NLP. Among them, 18 patients received intramuscular injections of triamcinolone acetonide (0.5 to 1 mg/kg) for a total of 3 to 6 months of treatment. Of these, 2 patients achieved mild improvement, 4 showed moderate improvement, 4 demonstrated great improvement, and 8 achieved clinical cure. An additional 12 patients were treated with oral methylprednisolone at a dose of 0.5 mg/kg. Three of these patients discontinued treatment after 2 months due to adverse effects, with 2 showing no clinical benefit and 1 achieving only minimal improvement. Among the remaining 9 patients who completed 6 to 12 months of treatment, 1 experienced moderate improvement, 2 achieved great improvement, and 6 reached clinical cure. However, disease relapse occurred in 6 of the 14 patients (42.86%) who initially achieved clinical cure within 6 months of treatment cessation. In patients with moderate to severe classical NLP, we found that there was no statistically significant difference in treatment response between oral methylprednisolone and intramuscular triamcinolone acetonide (*p* = 0.91). Interestingly, a significantly better response to systemic corticosteroid treatment was observed in patients with periungual erythema compared to those without (*p* < 0.05). In terms of safety, common adverse events included weight gain, transient cushingoid features, mild mood changes, and gastrointestinal symptoms. No severe complications were reported, such as avascular necrosis of the femoral head.

#### 3.9.4 JAK Inhibitor

One patient with severe classical NLP received oral Baricitinib with great improvement after 6 months. Another 2 patients (previous published [11, 12]), one with moderate classical NLP and another with severe classical NLP, received oral Baricitinib and Abrocitinib separately and achieved near‐complete remission after 6 months of treatment. In the 6‐month follow‐up, one of them experienced a minimal relapse. In terms of safety, no severe adverse events were reported. Laboratory monitoring during treatment revealed no clinically significant abnormalities in liver function, lipid profiles, or blood counts.

## 4 Discussion

Our findings demonstrate several epidemiological patterns that align with previous studies conducted in Western populations. The mean age of NLP onset was 44.41 ± 17.77 years, with a wide range extending from 6 to 75 years. This broad age spectrum aligns with previous reports, confirming that NLP can affect individuals across the lifespan, although the peak incidence tends to occur in middle‐aged adults [5, 6, 7, 8, 9]. Pediatric involvement was relatively uncommon in our series (11.11%), consistent with existing literature in which childhood NLP is reported to be rare and often underdiagnosed due to atypical presentations [5]. The observed male predominance (M:F ratio 1.45) in our study is consistent with prior findings, including the cohort described by Goettmann et al. [6], who reported a male‐to‐female ratio of 1.8 in 67 patients with NLP. This suggests that NLP may have a slight male predominance.

Clinically, we found that nail matrix involvement was overwhelmingly dominant, affecting 97.53% of cases, with classical matrix changes such as longitudinal ridging, splitting, and nail thinning being the most prevalent findings. This is consistent with the known pathophysiology of NLP, which predominantly targets the matrix [10]. Besides, our data also revealed that isolated toenail involvement was rare (2.47%), and the majority of patients (69.14%) had disease limited to the fingernails, supporting previous observations that fingernails are more frequently affected [6]. 9.88% of patients (*n* = 8) presented with NLP limited to a single fingernail, which is often under‐recognized. Moreover, our study found that patients with severe disease tended to have a longer disease course compared to those with mild or moderate involvement; the difference did not reach statistical significance. This finding is consistent with the unpredictable progression of NLP and highlights that even patients with relatively short disease duration may develop severe manifestations.

All 81 cases were confirmed histologically through longitudinal biopsies, except for the characteristic findings as previously mentioned. One of the most novel findings in our series was the frequent histopathological involvement of the ventral proximal nail fold (PNF), particularly at its junction with the proximal nail matrix. Although these changes were not evident on clinical examination, they were observed in over half of the patients (*n* = 47, 58.02%). While no statistically significant correlation was found between PNF involvement and disease severity, this finding highlights a previously underrecognized site of inflammation in NLP. As the ventral PNF plays a critical role in shielding and regulating the nail matrix microenvironment, its involvement may have pathogenic significance, potentially contributing to matrix inflammation.

The high diagnostic yield of our approach is further supported by the 96.30% concordance rate between clinical and histopathologic localization. This validates the utility of longitudinal nail biopsy as the gold standard for comprehensive histopathologic assessment in NLP, which can capture multiple compartments of the nail unit [10]. However, the benefit of diagnostic accuracy must be weighed against potential procedural morbidity. In our study, we found 14.52% of the following patients reported dissatisfaction (score < 6), predominantly due to postoperative nail deformities, which was the main concern of longitudinal biopsy [13]. To mitigate cosmetic sequelae, several technical strategies should be considered. In our practice, lateral matrix sampling was prioritized to preserve central nail growth. Careful site selection, atraumatic tissue handling, and fine suture closure were employed to minimize disruption of the nail architecture. Pre‐procedural counseling was also emphasized to align patient expectations and improve satisfaction. Given these considerations, we advocate for the judicious use of longitudinal biopsy, reserving it for cases with diagnostic uncertainty, atypical presentations, or when confirmation is essential to guide long‐term therapy.

A striking finding was the high rate of misdiagnosis and mistreatment prior to referral, with 44.44% of patients initially misdiagnosed. This may be attributed to the nonspecific and variable clinical presentations of NLP, which can mimic other nail disorders such as onychomycosis, psoriatic nail disease, or nonspecific dystrophies [10].

Recent expert consensus on the treatment of NLP suggests that topical therapies provide limited short‐term efficacy and may pose risks of long‐term adverse effects [3]. Instead, intralesional or intramuscular triamcinolone acetonide is recommended as a first‐line treatment, and oral retinoids are considered second‐line options, while immunosuppressive agents may be reserved for refractory cases [3]. Consistent with these recommendations, our findings confirmed that topical therapies alone, including high‐potency corticosteroids and calcineurin inhibitors, demonstrated minimal clinical benefit. Oral acitretin yielded modest efficacy, with more favorable outcomes observed in early‐stage or mild NLP. Hydroxychloroquine, despite its use in mucosal and cutaneous LP, was ineffective in all four treated patients, aligning with previously reported data. In contrast, systemic corticosteroids were the most effective treatment option in our cohort, particularly in classical NLP with active disease. However, their long‐term use was limited by treatment‐related adverse effects and a substantial relapse rate of 42.86% in patients who initially achieved clinical cure. Given the limitations of long‐term systemic corticosteroid use, alternative delivery strategies such as intralesional injections may help reduce systemic exposure, though their use is technically demanding, especially in cases with multiple nail involvement.

Interestingly, patients exhibiting periungual inflammation had a significantly better response to systemic corticosteroids compared to those without such findings (*p* < 0.05). This observation suggests that nail fold inflammation may reflect active, reversible inflammation, potentially serving as a clinical indicator of steroid responsiveness; though prospective validation is warranted.

The use of JAK inhibitors represents a novel and mechanistically targeted approach for the treatment of NLP [14]. The efficacy of JAK inhibitor for NLP can be explained by its blockade of the IFN‐γ–driven JAK–STAT signaling axis, which is considered central to LP pathogenesis [15]. Although clinical data on JAK inhibition for NLP remain limited, case reports have documented encouraging outcomes using JAK inhibitors, all reporting significant efficacy and tolerability [16, 17, 18]. In our cohort, one patient achieved near‐complete remission after 6 months of Baricitinib monotherapy, while two previously reported patients experienced similar responses to either Baricitinib or Abrocitinib. Given the limitations of current systemic therapies for NLP, including corticosteroid‐related side effects and high relapse rates, JAK inhibitors offer a promising alternative, particularly for severe or treatment‐refractory cases. Selective JAK inhibitors may further provide a more favorable safety profile compared to pan‐JAK blockade; though prospective trials are needed to confirm these early findings and establish optimal treatment protocols for NLP.

This study also has several limitations. First, the classification of disease severity and treatment response evaluation were based on the criteria proposed by Iorizzo et al. [3], which are inherently subjective and lack a quantitative scoring system. Second, due to the small sample size and clinical heterogeneity, we were unable to perform advanced statistical analyses such as Kaplan–Meier survival analysis, Cox proportional hazards modeling, or logistic regression; further stratified analyses of treatment efficacy and response duration across different therapies were not conducted. Third, the cohort lacked sufficient cases of intralesional corticosteroid therapy, limiting the assessment of its efficacy. Additionally, long‐term follow‐up data were not available for all patients. Lastly, the retrospective design may introduce selection and recall bias. Future prospective studies with larger cohorts and standardized scoring systems are necessary to validate and expand upon our findings.

In conclusion, we provide a comprehensive analysis of the clinical, histopathological, and therapeutic features of NLP. Our findings highlight the predominant involvement of the nail matrix, emphasize the diagnostic value of longitudinal nail biopsies in identifying subclinical inflammation in the ventral proximal nail fold, and reveal a substantial rate of prior misdiagnosis. While systemic corticosteroids were most effective, especially in cases with periungual erythema, relapse was common. Promising responses to JAK inhibitors suggest potential new treatment avenues. We believe that these insights may lay the groundwork for future studies.

## Ethics Statement

The study received approval from the ethical committee (S2023‐179) of the Chinese PLA General Hospital.

## Consent

The patients in this manuscript have given written informed consent to the publication of their case details.

## Conflicts of Interest

The authors declare no conflicts of interest.

## Data Availability

The data that support the findings of this study are available on request from the corresponding author. The data are not publicly available due to privacy or ethical restrictions.
